# Supplementary material for: Association between continuous glucose monitoring-derived metrics and coronary plaque vulnerability: A retrospective exploratory analysis
Source: eLife. 2026 Jan 15;14:RP102860. doi: 10.7554/eLife.102860 (PMC12807456; doi:10.7554/eLife.102860)
Supplement: Supplementary file 1. [file elife-102860-supp1.docx]

**Supplementary File 1. Calculating formulae of the continuous glucose monitoring-derived indices.**

| Name | Formulae | Variables |
| --- | --- | --- |
| CGM_Mean | $\frac{1}{N}\sum_{n=1}^{N} G_{n}$ | $G=$glucose measured  $N=$total number of readings |
| CGM_Std | $\sqrt{\frac{1}{N-1}\sum_{n=1}^{N} {(G}_{n}-\bar{G})}$ | $G=$glucose measured  $N=$total number of readings |
| CONGA | $\sqrt{\frac{1}{k-1}\sum_{t=t_{1}}^{t_{k}} {(D_{t}-\bar{D})}^{2}}$  $\bar{D}=\frac{1}{k}\sum_{t=t_{1}}^{t_{k}} D_{t}, D_{t}=G_{t}-G_{t-60}$ | $G=$glucose measured  $k=$number of observations with an observation 60 min ago  $t=$time |
| LI | $\sum_{n=1}^{N-1} \frac{{(G_{n}-G_{n+1})}^{2}}{t_{n+1}-t_{n}}$ | $G=$glucose measured  $N=$total number of readings  $t=$time |
| JINDEX | $0.324\times{(CGM\_Mean+CGM\_Std)}^{2}$ |  |
| HBGI | $\frac{1}{N}\sum_{i=1}^{N} {\mathrm{rh}(x}_{i})$ | $x=$nonlinear transformation of glucose measured  $N=$total number of readings  $\mathrm{rh}=$risk value associated with a high glucose |
| GRADE | $\mathrm{median}{(425\times\left\{ log\left[ log(G_{n}) \right]+0.16 \right\}}^{2})$ | $G=$glucose measured |
| MODD | $\frac{1}{k}\sum_{t=t_{1}}^{t_{k}} \left\vert G_{t}-G_{t-1440} \right\vert$ | $G=$glucose measured  $k=$number of observations with an observation 24 h ago  $t=$time |
| MAGE | $\sum\frac{\lambda}{x} if \lambda>v$ | $\lambda=$blood glucose changes from peak to nadir  $x=$number of observations  $v=$1 Std of mean glucose for 24 h period |
| ADRR | $\frac{1}{N}\sum_{n=1}^{N} \left[ \mathrm{LR}+\mathrm{HR} \right]$ | $N=$total number of readings  $\mathrm{LR}=$risk value attributed to low glucose  $\mathrm{HR}=$risk value attributed to high glucose |
| M-value | $\frac{1}{N}\sum_{t=t_{1}}^{t_{k}} \left\vert10log\frac{{18G}_{t}}{\mathrm{IGV}} \right\vert^{3}$ | $G=$glucose measured  $k=$number of observations IGV=ideal glucose value  $t=$time |
| MAG | $\frac{1}{T}\sum_{n=1}^{N-1} {(G}_{n}-G_{n+1})$ | $G=$glucose measured  $N=$total number of readings  $T=$total time |
| AC_Mean | $\frac{1}{30}\sum_{l=1}^{30} \mathrm{AC}_{l}$ | $\mathrm{AC}=$autocorrelation of glucose  $l=$lag |
| AC_Var | $\frac{1}{29}\sum_{l=1}^{30} {{(\mathrm{AC}}_{l}-AC\_Mean)}^{2}$ | $\mathrm{AC}=$autocorrelation of glucose  $l=$lag |
